# Enhancing Thermal Oxidation Stability of Silver Nanowire Transparent Electrodes by Using a Cesium Carbonate-Incorporated Overcoating Layer

**DOI:** 10.3390/ma12071140

**Published:** 2019-04-08

**Authors:** Yong-Chan Jeong, Jiyoon Nam, Jongbok Kim, Chang Su Kim, Sungjin Jo

**Affiliations:** 1 Advanced Functional Thin Films Department, Korea Institute of Material Science (KIMS), Changwon 51508, Korea; jansuk12@kims.re.kr; 2 Department of Materials Science and Engineering, Kumoh National Institute of Technology, Gumi 39177, Korea; 3 School of Architectural, Civil, Environmental, and Energy Engineering, Kyungpook National University, Daegu 41566, Korea; sculptor_nj@naver.com

**Keywords:** Ag nanowire, thermal stability, oxidation stability, overcoating layer, cesium carbonate

## Abstract

Despite their excellent electrical and optical properties, Ag nanowires (NWs) suffer from oxidation when exposed to air for several days. In this study, we synthesized a Cs carbonate-incorporated overcoating layer by spin-coating and ultraviolet curing to prevent the thermal oxidation of Ag NWs. Cs incorporation increased the decomposition temperature of the overcoating layer, thus enhancing its thermal resistance. The effects of the Cs carbonate-incorporated overcoating layer on the optoelectrical properties and stability of Ag NWs were investigated in detail. The Ag NW electrode reinforced with the Cs carbonate-incorporated overcoating layer exhibited excellent thermal oxidation stability after exposure to air for 55 days at 85 °C and a relative humidity of 85%. The novel overcoating layer synthesized in this study is a promising passivation layer for Ag NWs against thermal oxidation under ambient conditions. This overcoating layer can be applied in large-area optoelectronic devices based on Ag NW electrodes.

## 1. Introduction

Ag nanowire (NW) transparent electrodes are potential alternatives to indium tin oxide transparent conductive electrodes and have been widely used as an essential element of various optoelectronic devices. Ag NW electrodes can be fabricated using low-cost solution processes such as bar-coating, spin-coating, and spray-coating [1,2,3]. In addition, when deposited on flexible substrates, they can maintain their conductivity during repeated bending. Hence, Ag NW electrodes have been used as transparent and flexible electrodes in a wide range of devices such as displays, photovoltaics, touch panels, and thin film heaters [4,5,6,7]. 

Despite the excellent properties mentioned above, Ag NW electrodes suffer from poor thermal oxidation stability, which degrades their electrical and optical properties during post-manufacturing processes or in actual operation. In order to overcome this limitation, various overcoating layers have been developed to prevent the oxidation of Ag NWs, and hence to enhance the long-term stability of Ag NW electrodes. For example, Lee et al. used chemical vapor deposition-grown monolayer graphene as a protection layer [8], whilst Hwang et al. used Al_2_O_3_ formed by atomic layer deposition as an ultrathin encapsulation layer [9]. In addition, Chen et al. developed a neutral-pH poly(3,4-ethylenedioxythiophene): poly(styrenesulfonate) overcoating layer [10]. A more detailed comparison with previous works can be found in Table S1. However, the development of a highly efficient passivation layer that can not only be applied to large areas uniformly but can also protect Ag NWs from oxidation is challenging. Hence, it is imperative to develop a facile method to synthesize such passivation layers in order to broaden the application of Ag NW electrodes to various flexible devices. 

In this study, we synthesized a cesium carbonate (Cs_2_CO_3_)-incorporated overcoating layer, which exhibited excellent thermal oxidation stability. The alkali carbonates are fairly stable at a high temperature, thereby offering a high thermal stability of the overcoating layer. Moreover, this overcoating layer could be developed by simple spin-coating and ultraviolet (UV) curing processes, which are feasible for large-scale roll-to-roll processes. The effect of the Cs_2_CO_3_ content on the thermal oxidation stability of the Ag NW electrodes was investigated by determining their transmittance, haze, and sheet resistance values. In addition, the efficacy of the Cs_2_CO_3_-incorporated overcoating layer was demonstrated by exposing the Ag NW electrodes to air at 85 °C and a relative humidity of 85% for 55 days. 

## 2. Materials and Methods

### 2.1. Ag NW Electrode Fabrication and Characterization

Ag NWs dispersed in deionized water were purchased from C3Nano Co., Ltd. (Hayward, USA). The average length and diameter of the Ag NWs were approximately 25 μm and 30 nm, respectively. The Ag NW suspension was spin-coated on a polyether sulfone (PES) substrate at 3000 rpm and then annealed at 130 °C for 1 min to completely evaporate the solvent. The thickness of the Ag NW film was ~60 nm. The transmittance values of the resulting Ag NW electrodes were determined using a UV-visible (UV-Vis) spectrometer (Agilent Technologies) (Santa Clara, USA). The sheet resistances (*R_s_*) of the electrodes were measured using a four-point probe system (FPP-2400) (DASOLENG, Chungju, Korea). The surface morphologies of the Ag NW electrodes were examined using field emission scanning electron microscopy (FE-SEM, JSM-6700F) (JEOL, Tokyo, Japan). and atomic force microscopy (AFM, NX10) (Park System, Suwon, Korea). The ambient stability of the electrodes was evaluated by exposing them to an air environment at 85 °C and a relative humidity of 85% in a thermo-hygrostat. 

### 2.2. Fabrication of the UV-Curable Passivation Layer

Silica Sol monomer (ASAM-6100) was provided by Ranco (Jincheon-Gun, Korea). 1-Hydroxycyclohexyl-phenyl-ketone (Irgacure 184, 99%) and diphenyl(2,4,6-trimethylbenzoyl)-phosphine oxide (TPO, 97%), used as photoinitiators, were purchased from Sigma-Aldrich (St. Louis, USA). The silicone-containing surface additive (BYK-310) was supplied by BYK (Wesel, Germany). For the preparation of the passivation layer, first, ASAM-6100 (1 wt%), Irgacure 184 (0.3 wt%), and TPO (0.1 wt%) were added to a solution of isopropyl alcohol (IPA) and diacetone alcohol. Then, 0.1 wt% BYK-310 was added, and the resulting mixture was stirred vigorously at 25 °C. To fabricate the alkali metal carbonate-incorporated overcoating layers, 0.05 wt% of various alkali metal carbonate powders (K_2_CO_3_, Rb_2_CO_3_, and Cs_2_CO_3_) was added to the mixture. In this case, ethanol was used as the solvent instead of IPA. The overcoating layer was spin-coated at 1000 rpm followed by annealing at 80 °C for 1 min to remove the solvent. The overcoating layer deposited on the Ag NW electrodes was then cured by exposing it to UV light for 1 min (Figure S1).

## 3. Results and Discussion

**Figure 1:**
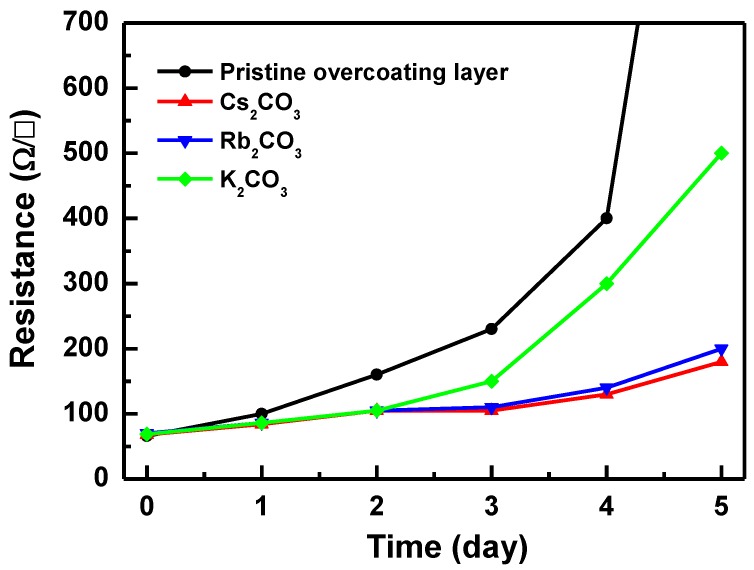
Sheet resistance of Ag nanowires (NWs) covered with pristine and various alkali carbonate-incorporated overcoating layers annealed at 150 °C for 5 days.

The effect of the alkali metal carbonates on the thermal stability of the Ag NW electrodes was investigated by measuring their *R_s_* values at 150 °C for 5 days (Figure 1). The *R_s_* of the Ag NW electrode covered with the pristine overcoating layer increased significantly after 4 days. The *R_s_* of the Ag NWs covered with the K_2_CO_3_-incorporated overcoating layer increased up to 500 ohm/sq. The Ag NW electrodes covered with the Cs_2_CO_3_- and Rb_2_CO_3_-incorporated overcoating layers showed an *R_s_* of less than 200 ohm/sq. Although the K and Rb carbonate-incorporated layers showed higher thermal resistance than the pristine overcoating layer, the thermal resistance of the Cs_2_CO_3_-incorporated overcoating layer was found to be optimum. The increase in the resistivity of the Ag NWs was caused by accelerated surface diffusion of atoms because of their high Gibbs–Thomson potential gradient [11]. As a result, the NWs became unstable under thermal stress at a temperature significantly lower than the melting temperature of bulk Ag. Hence, it can be stated that the deposition of Cs_2_CO_3_-incorporated overcoating layers effectively prevents the surface diffusion of Ag atoms, thereby enhancing the thermal stability of Ag NWs [12].

**Figure 2:**
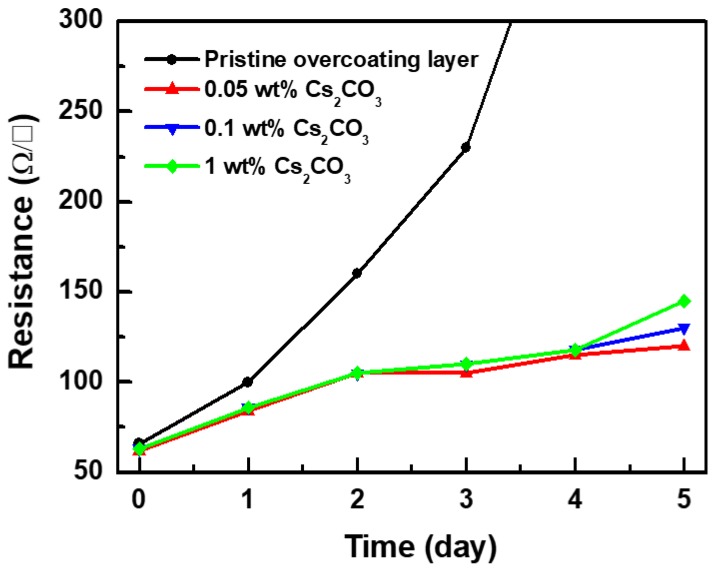
Sheet resistance of Ag NWs covered with pristine and Cs_2_CO_3_-incorporated overcoating layers annealed at 150 °C for 5 days.

The effect of the Cs_2_CO_3_ content on the properties of the Cs_2_CO_3_-incorporated overcoating layer was investigated (Figure 2). The Cs_2_CO_3_-incorporated overcoating layers showed excellent heat-resistant properties irrespective of their Cs_2_CO_3_ contents. However, the overcoating layer containing 1 wt% Cs_2_CO_3_ showed a significantly high haze value because of the presence of undissolved Cs_2_CO_3_ (Table S1). The overcoating layer with 0.05 wt% Cs_2_CO_3_ showed the best heat resistance and optical properties. Hence, the Cs_2_CO_3_ content of 0.05 wt% was found to be optimum.

To quantitatively analyze the effect of the Cs_2_CO_3_-incorporated overcoating layer on the optoelectrical properties of the Ag NW electrode, its transmittance and *R_s_* values were measured before and after the deposition of the overcoating layer. The transmittance and *R_s_* values of the uncoated Ag NW electrode (deposited on a PES substrate) were 87% and 70 ohm/sq, respectively. The Ag NW electrode maintained its electrical properties even after the deposition of the overcoating layer, indicating that the overcoating layer did not significantly alter the electrical properties of the Ag NWs (Table S2). On the other hand, the transmittance of the Ag NW electrode increased slightly after the deposition of the overcoating layer (Figure S2). Since the dielectric environment in which Ag NWs are embedded affects their optical properties, the graded index refraction of the overcoating layer from the air to the substrate can suppress their reflectance [13].

**Figure 3:**
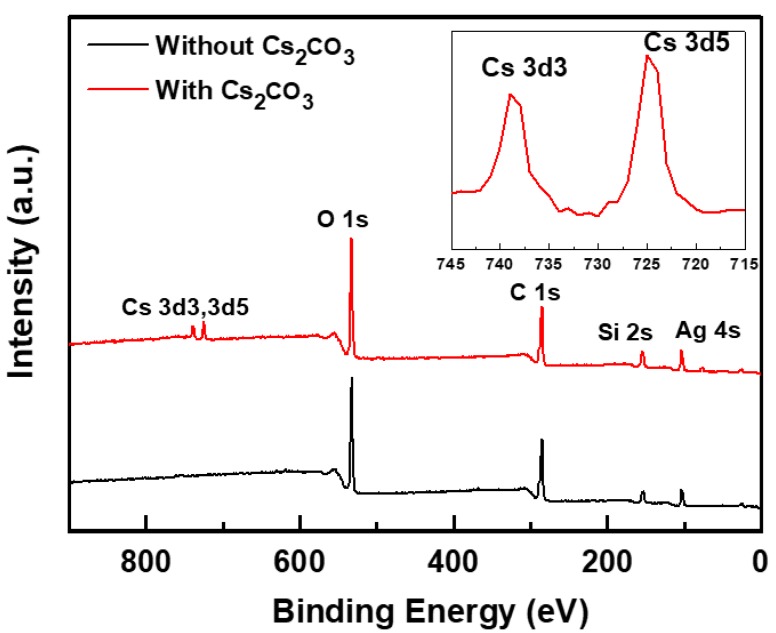
X-ray photoelectron spectroscopy (XPS) spectra of overcoating layers with and without Cs_2_CO_3_. Inset shows the Cs 3d3 and Cs 3d5 spectra of the overcoating layers with and without Cs_2_CO_3_.

X-ray photoelectron spectroscopy (XPS) was used to confirm the incorporation of Cs_2_CO_3_ in the overcoating layer. Figure 3 shows the overall XPS peaks of the overcoating layers with and without Cs_2_CO_3_. The Cs_2_CO_3_-incorporated overcoating layer showed Cs 3d3 and Cs 3d5 peaks at 739 and 726 eV, respectively, confirming the presence of Cs within the layer [14]. 

**Figure 4:**
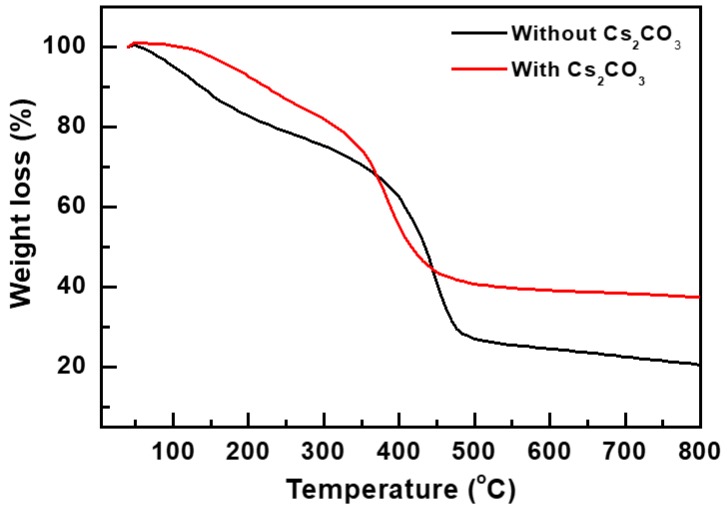
Thermogravimetric analysis (TGA) curves of overcoating layers with and without Cs_2_CO_3_.

The thermal degradation characteristics of the overcoating layers were investigated by carrying out their thermogravimetric analysis (TGA) by heating them from 25 to 800 °C at a rate of 10 °C/min. The TGA curves of the overcoating layers are shown in Figure 4. The decomposition onset temperatures of the overcoating layers with and without Cs_2_CO_3_ were 173 and 104 °C, respectively. This indicates that the addition of Cs_2_CO_3_ enhanced the heat resistance of the overcoating layer. Hence, the degradation of the overcoating layer without Cs_2_CO_3_ resulted in a rapid increase in the *R_s_* of the corresponding Ag NW electrode. The thermal degradation of polymers occurs by the loss of hydrogen atoms. This degradation propagates when free radicals react with oxygen molecules to form hydroperoxides [15]. Although the thermal stability enhancement mechanism of Cs_2_CO_3_-incorporated overcoating layers is not clear at present, it can be attributed to the ability of Cs to prevent the oxidation of free radicals of polymer molecules. 

**Figure 5:**
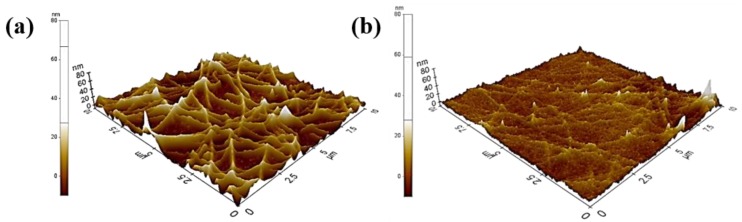
Atomic force microscopy (AFM) images of (**a**) pristine Ag NWs and (**b**) Ag NWs covered with the Cs_2_CO_3_-incorporated overcoating layer.

The surface roughness as well as the optoelectrical properties of Ag NW electrodes are important. This is because high peak-to-valley values can cause an electrical short-circuit or a current leakage, which hinders the efficiency of the optoelectronic device [16]. Figure 5a,b show the AFM images of the Ag NW electrodes without and with the Cs_2_CO_3_-incorporated overcoating layer, respectively. The roughness value (R_a_) of the Ag NW electrode without the overcoating layer was 6.933 nm, whereas that of the Ag NW electrode with the overcoating layer was 2.902 nm. This indicates that the spin-coated overcoating layer thoroughly permeated the Ag NW network and filled the voids within it. 

**Figure 6:**
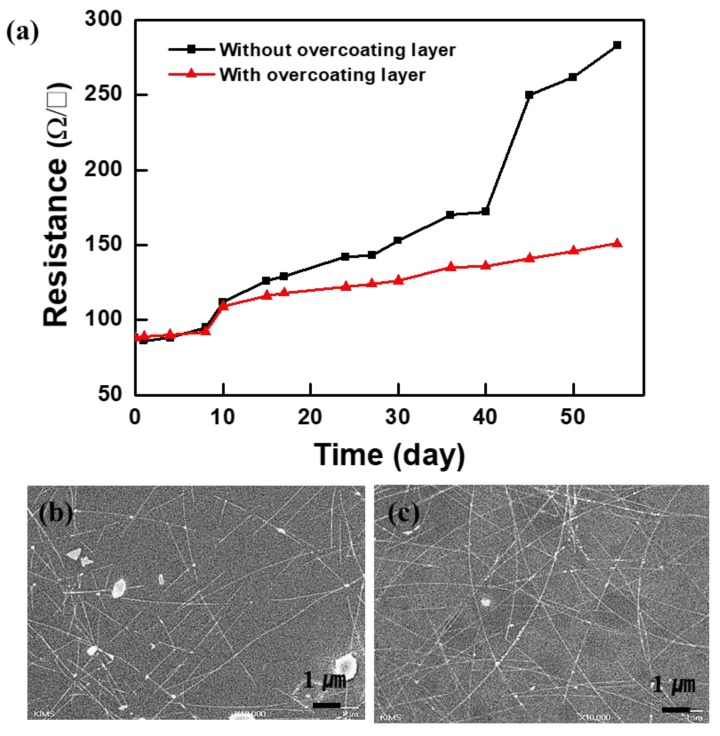
(**a**) Thermal resistance of Ag NWs exposed at 85 °C and a relative humidity of 85% for 55 days. Scanning electron microscopy (SEM) images of Ag NWs after exposure for 55 days: (**b**) pristine Ag NWs and (**c**) Ag NWs with the overcoating layer.

To investigate the effect of the overcoating layer on the thermal oxidation stability of the Ag NW electrodes, we determined their *R_s_* values before and after the deposition of the overcoating layer at 85 °C and a relative humidity of 85% for 55 days. As shown in Figure 6a, the *R_s_* value of the pristine Ag NWs increased drastically after 40 days. This can be attributed to the facile oxidation of Ag NWs upon exposure to humid air because of their large surface-to-volume ratios [17]. On the other hand, the *R_s_* of the Ag NW electrode covered with the Cs_2_CO_3_-incorporated overcoating layer increased slightly until day 55. To further investigate the origin of the improved long-term stability, we investigated the surface morphologies of the electrodes. The Ag NW electrodes were placed in a thermo-hygrostat for 55 days. Figure 6b,c show the scanning electron microscopy (SEM) images of the Ag NW electrodes with and without the overcoating layer, respectively. The Ag NW electrode covered with the overcoating layer showed a relatively smooth surface and small protrusions. On the other hand, in the case of the electrode without the overcoating layer, the Ag NWs broke at the protrusions. The high *R_s_* of the Ag NW electrode without the overcoating layer can be attributed to the connectivity loss between the nanowires in this case. 

## 4. Conclusions

In this study, we evaluated the ability of Cs_2_CO_3_ to prevent the thermal oxidation of Ag NWs. The Ag NWs encapsulated with the Cs_2_CO_3_-incorporated overcoating layer showed excellent thermal stability. The TGA results revealed that the addition of Cs_2_CO_3_ enhanced the thermal resistance of the overcoating layer, which likely prevented the oxidation of free radicals from the polymer molecules. In addition, the overcoating layer effectively blocked the diffusion of H_2_O molecules, which successfully prevented the oxidation of the Ag NWs when exposed to high humidity at elevated temperatures for a long duration (55 days). Moreover, the deposition of the Cs_2_CO_3_-incorporated overcoating layer decreased the surface roughness of the Ag NW electrode, thus preventing the optoelectronic device from electrical short-circuit or current leakage. Since Cs_2_CO_3_-incorporated overcoating layers can be deposited by a simple spin-coating process at low temperatures, they will have a great advantage over other overcoating layers for large-area optoelectronic devices based on Ag NW electrodes.

## Supplementary Materials

The following are available online at https://www.mdpi.com/1996-1944/12/7/1140/s1.

## References

[1] Zhang R., Engholm M. (2018). Recent progress on the fabrication and properties of silver nanowire-based transparent electrodes. Nanomaterials.

[2] Ye T., Jun L., Kun L., Hu W., Ping C., Ya-Hui D., Zheng C., Yun-Fei L., Hao-Ran W., Yu D. (2017). Inkjet-printed Ag grid combined with Ag nanowires to form a transparent hybrid electrode for organic electronics. Org. Electron..

[3] Choi Y., Kim C., Jo S. (2018). Spray deposition of Ag nanowire–graphene oxide hybrid electrodes for flexible polymer–dispersed liquid crystal displays. Materials.

[4] Canlier A., Ucak U.V., Usta H., Cho C., Lee J., Sen U., Citir M. (2015). Development of highly transparent Pd-coated Ag nanowire electrode for display and catalysis applications. Appl. Surf. Sci..

[5] Kim Y., Kim J. (2016). Silver nanowire networks embedded in urethane acrylate for flexible capacitive touch sensor. Appl. Surf. Sci..

[6] Nam S., Song M., Kim D., Cho B., Lee H.M., Kwon J., Park S., Nam K., Jeong Y., Kwon S. (2014). Ultrasmooth, extremely deformable and shape recoverable Ag nanowire embedded transparent electrode. Sci. Rep..

[7] Ha B., Jo S. (2017). Hybrid Ag nanowire transparent conductive electrodes with randomly oriented and grid-patterned Ag nanowire networks. Sci. Rep..

[8] Lee D., Lee H., Ahn Y., Jeong Y., Lee D., Lee Y. (2013). Highly stable and flexible silver nanowire–graphene hybrid transparent conducting electrodes for emerging optoelectronic devices. Nanoscale.

[9] Hwang B., An Y., Lee H., Lee E., Becker S., Kim Y., Kim H. (2017). Highly Flexible and Transparent Ag Nanowire Electrode Encapsulated with Ultra-Thin Al_2_O_3_: Thermal, Ambient, and Mechanical Stabilities. Sci. Rep..

[10] Chen S., Song L., Tao Z., Shao X., Huang Y., Cui Q., Guo X. (2014). Neutral-pH PEDOT: PSS as over-coating layer for stable silver nanowire flexible transparent conductive films. Org. Electron..

[11] Chen D., Liang J., Liu C., Saldanha G., Zhao F., Tong K., Liu J., Pei Q. (2015). Thermally stable silver nanowire–polyimide transparent electrode based on atomic layer deposition of zinc oxide on silver nanowires. Adv. Funct. Mater..

[12] Ahn Y., Jeong Y., Lee Y. (2012). Improved thermal oxidation stability of solution-processable silver nanowire transparent electrode by reduced graphene oxide. ACS Appl. Mater. Interfaces.

[13] Zhao Z., Wang K.X., Fan S. (2017). Analysis of an anti-reflecting nanowire transparent electrode for solar cells. J. Appl. Phys..

[14] Dong H., Guo X., Li W., Wang L. (2014). Cesium carbonate as a surface modification material for organic–inorganic hybrid perovskite solar cells with enhanced performance. RSC Adv..

[15] Xue T.J., McKinney M.A., Wilkie C.A. (1997). The thermal degradation of polyacrylonitrile. Polym. Degrad. Stab..

[16] Khaligh H.H., Goldthorpe I.A. (2014). Hot-rolling nanowire transparent electrodes for surface roughness minimization. Nanoscale Res. Lett..

[17] Chen J., Ahn H., Yen S., Tsai Y. (2014). Thermally induced percolational transition and thermal stability of silver nanowire networks studied by THz spectroscopy. ACS Appl. Mater. Interfaces.

