# Supplementary material for: Enhancing Thermal Oxidation Stability of Silver Nanowire Transparent Electrodes by Using a Cesium Carbonate-Incorporated Overcoating Layer
Source: Materials (Basel). 2019 Apr 8;12(7):1140. doi: 10.3390/ma12071140 (PMC6480286; doi:10.3390/ma12071140)
Supplement: Supplementary file 1 [file materials-12-01140-s001.pdf]

Supporting Information

Enhancing thermal oxidation stability of silver nanowire transparent electrodes by using a cesium carbonate-incorporated overcoating layer

Yong-Chan Jeong [1,2], Jiyoon Nam [3], Jongbok Kim [2,*], Sungjin Jo [3,*], Chang Su Kim [1,*]

[1]Advanced Functional Thin Films Department, Korea Institute of Material Science (KIMS), Changwon 51508, Korea

[2]Department of Materials Science and Engineering, Kumoh National Institute of Technology, Gumi 39177, Korea

[3]School of Architectural, Civil, Environmental, and Energy Engineering, Kyungpook National University, Daegu 41566, Korea

* Correspondence: jbkim@kumoh.ac.kr; Tel.: +82-54-478-7748, sungjin@knu.ac.kr; Tel.: +82-53-950-8971, cskim1025@kims.re.kr; Tel. :+82-55-280-3696

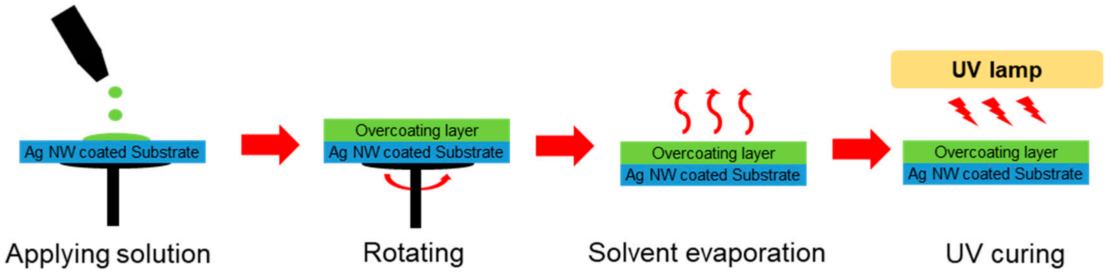

Figure S1. Schematic illustration of the steps involved in fabricating the overcoating layer.

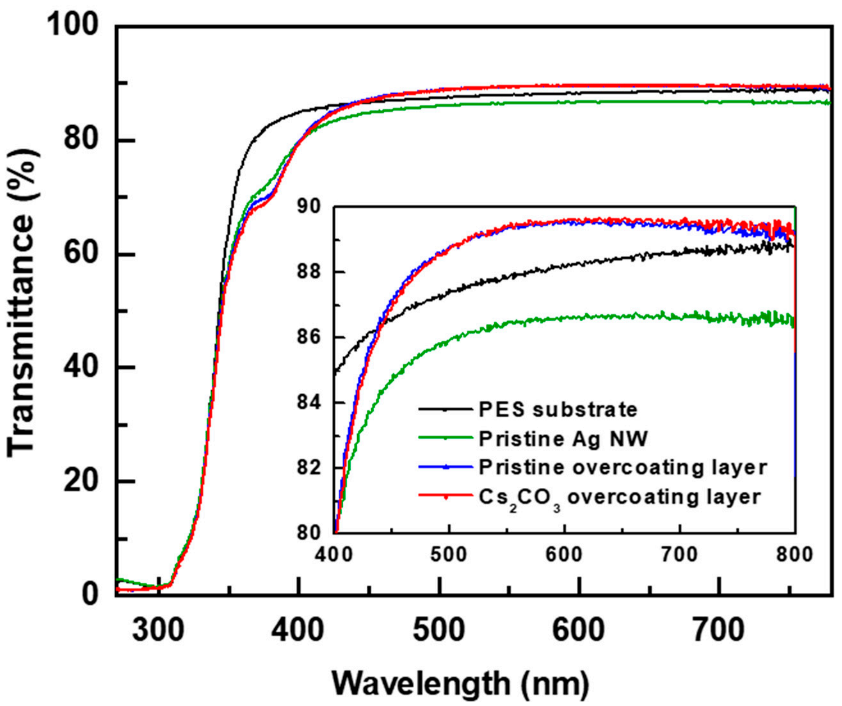

Figure S2. Optical transmission spectra of the PES substrate, pristine Ag NWs, Ag NWs covered with the pristine overcoating layer, and Ag NWs covered with the Cs_2CO_3-incorporated overcoating layer. Inset shows the magnified optical transmission spectra of the samples from 80% to 90% transmittance.

Table 1. Comparison of our experimental results with previous reports.

**Reference**	**Overcoating material**	**Deposition method**	**Ambient stability test**
Lee et al. [1]	Graphene	Transfer printing	70 °C, 70% RH, 8 days
Hwang et al. [2]	Al_2O_3	ALD	380 °C, 100 min
Chen et al. [3]	PEDOT:PSS	Spin coating	Air, 108 h
Chen et al. [4]	ZnO	ALD	300 °C, 6 h
Ahn et al. [5]	Reduced graphene oxide	Dip coating	70 °C, 70% RH, 8 days
This work	Cs_2CO_3 incorporated layer	Spin coating	85 °C, 85% RH, 55 days

Table S2. Optical transmittance, sheet resistance, and haze values of the samples.

	Transmittance (%)	Sheet resistance (ohm/□)	Haze
Pristine Ag NW	87.29	66	0.83
Pristine overcoating layer	89.90	66	0.77
Cs_2CO_3 0.05 wt% overcoating layer	89.95	61	0.88
Cs_2CO_3 0.1 wt% overcoating layer	89.79	64	0.82
Cs_2CO_3 1 wt% overcoating layer	89.58	63	5.12

References

[1] Lee, D.; Lee, H.; Ahn, Y.; Jeong, Y.; Lee, D.; Lee, Y. Highly stable and flexible silver nanowire–graphene hybrid transparent conducting electrodes for emerging optoelectronic devices. *Nanoscale* **2013,** 5, 7750-7755.

[2] Hwang, B.; An, Y.; Lee, H.; Lee, E.; Becker, S.; Kim, Y.; Kim, H. Highly Flexible and Transparent Ag Nanowire Electrode Encapsulated with Ultra-Thin Al_2O_3: Thermal, Ambient, and Mechanical Stabilities. *Sci. Rep.* **2017,** 7, 41336.

[3] Chen, S.; Song, L.; Tao, Z.; Shao, X.; Huang, Y.; Cui, Q.; Guo, X. Neutral-pH PEDOT: PSS as over-coating layer for stable silver nanowire flexible transparent conductive films. *Org. Electron.* **2014,** 15, 3654-3659.

[4] Chen, D.; Liang, J.; Liu, C.; Saldanha, G.; Zhao, F.; Tong, K.; Liu, J.; Pei,Q. Thermally stable silver nanowire–polyimide transparent electrode based on atomic layer deposition of zinc oxide on silver nanowires. *Adv. Funct. Mater.* **2015,** 25, 7512-7520.

[5] Ahn, Y.; Jeong, Y.; Lee, Y. Improved thermal oxidation stability of solution-processable silver nanowire transparent electrode by reduced graphene oxide. *ACS Appl. Mater. Inter.* **2012,** 4, 6410-6414.
